# Hereditary Predisposition to Acute Myeloid Leukemia: A Novel Germline CEBPA Mutation in a Multigenerational Family

**DOI:** 10.1155/crh/8189036

**Published:** 2026-09-27

**Authors:** Nancy Aislinn Tónix-Ramírez, Eneida Sánchez-Medina, Luis Enrique Muñóz-Pérez, Raquel Hernández-Ramos, Claudia Guevara-Espejel, Elizabeth Bautista-Rodríguez, Federico Osorio-Antonio

**Affiliations:** ^1^ Department of Pediatrics, Health Services of the Instituto Mexicano del Seguro Social Para el Bienestar, Children’s Hospital Tlaxcala, Tlaxcala 90606, Mexico; ^2^ Department of Pediatric Hematology, Health Services of the Instituto Mexicano del Seguro Social Para el Bienestar, Children’s Hospital Tlaxcala, Tlaxcala 90606, Mexico; ^3^ Department of Pediatric Oncology, Health Services of the Instituto Mexicano del Seguro Social Para el Bienestar, Children’s Hospital Tlaxcala, Tlaxcala 90606, Mexico; ^4^ Faculty of Health Sciences, Autonomous University of Tlaxcala, Tlaxcala 90606, Mexico, uatx.mx; ^5^ Department of Genetics, Health Services of the Instituto Mexicano del Seguro Social Para el Bienestar, Children’s Hospital Tlaxcala, Tlaxcala 90606, Mexico

**Keywords:** acute myeloid leukemia, CEBPA, germline predisposition, pediatric leukemia

## Abstract

Acute myeloid leukemia (AML) is a heterogeneous hematologic malignancy driven by the accumulation of genetic alterations in hematopoietic progenitor cells. CEBPA variants have been linked to familial AML, although multigenerational cases have been infrequently reported. We describe the case of a 6‐year‐old boy referred for hematologic abnormalities, with a history of idiopathic thrombocytopenic purpura at age 3. His family history revealed 11 cases of AML in four maternal generations, suggesting autosomal dominant inheritance. Diagnostic evaluation revealed AML with 40% bone marrow blasts, a normal karyotype, and a negative multiplex RT‐PCR panel for 28 common chromosomal translocations. Next‐generation sequencing (NGS) identified a novel heterozygous pathogenic variant in the CEBPA gene (c.273_309delinsGGCCAGGGTCT; p.Lys92Alafs∗7), which was predicted to generate a truncated protein. Given the patient’s medical history, genetic screening was performed on the patient’s first‐degree relatives. Genetic testing subsequently identified the same variant in the patient’s mother, supporting its germline origin, while his sister showed no alterations in the CEBPA gene sequence. The presence of the variant in the patient´s mother, who is asymptomatic, is consistent with autosomal dominant transmission and age‐dependent or incomplete penetrance. In this case, the patient carried a heterozygous variant, achieved complete remission after chemotherapy, and remains under surveillance. We present a pathogenic germline deletion‐insertion variant in the CEBPA gene, not previously described in the literature, associated with hereditary predisposition to AML in a four‐generation family, highlighting the variable expressivity characteristic of this syndrome and the relevance of considering a hereditary predisposition disorder in pediatric patients with AML.

## 1. Introduction

Acute myeloid leukemia (AML) has an incidence of approximately 4.3–5.4 cases per 100,000 people per year [1]. It predominantly affects adults, with an age‐adjusted incidence of 20.1 cases per 100,000 individuals aged 65 years or older. In the United States, approximately 75% of AML cases occur in adults older than 65 years [1]. In contrast, among children aged 0–14 years, AML accounts for 15%–20% of all leukemia cases [2, 3]. Prognosis varies considerably with age. The overall 5‐year survival rate is approximately 32%, with overall survival reported at approximately 50% in younger patients and up to 70% in pediatric patients, compared with approximately 10% in patients older than 60 years [4–6].

AML is a heterogeneous hematologic neoplasm characterized by the clonal proliferation and impaired differentiation of myeloid precursor cells in the bone marrow, generally driven by the accumulation of genetic alterations in hematopoietic progenitor cells [7]. Although AML has traditionally been considered predominantly sporadic, advances in sequencing technologies have improved understanding of its genetic basis and have contributed to advances in diagnosis, risk stratification, targeted therapy, and prognosis [8]. The sequential acquisition of somatic mutations in hematopoietic stem and progenitor cells plays a central role in AML pathogenesis. Approximately 40–50 genes have been identified as recurrently mutated in different types of leukemia, and their characterization has improved diagnosis, risk stratification, treatment selection, and prognostic assessment [7, 8]. Although molecular studies of leukemia have historically focused primarily on somatic alterations, germline variants that predispose individuals to hematologic malignancies are increasingly recognized and have been reported in up to 8% of pediatric and adult cases, with some leukemias showing familial aggregation [9].

Among the genes associated with hereditary predisposition to AML is CEBPA, which encodes the transcription factor CCAAT/enhancer‐binding protein alpha (C/EBPα), a key regulator of granulopoiesis. C/EBPα also interacts with CDK2 and CDK4 and contributes to the regulation of cell cycle progression, proliferation, and differentiation of myeloid progenitor cells [10]. Both somatic and germline CEBPA variants have been associated with AML. Germline pathogenic variants are associated with familial myeloid leukemia, typically showing autosomal dominant inheritance and an earlier age at onset than sporadic AML [11, 12]. Although hereditary predisposition associated with germline CEBPA variants has been previously described, reports involving multiple generations remain limited. Characterization of such families is essential to understand penetrance, variability in clinical expression among carriers, and the implications for genetic counseling and surveillance of asymptomatic relatives.

In this report, we present the clinical and molecular analysis of a family with AML spanning four maternal generations and a germline CEBPA deletion‐insertion (*delins*) variant not previously reported in the literature. We aimed to characterize the clinical presentation and family pattern, document germline transmission of the variant, and discuss its potential implications for genetic counseling, clinical management, and surveillance of at‐risk family members.

## 2. Case Report

A 6‐year‐old male patient was referred to the Hematology Department of Tlaxcala Children’s Hospital for evaluation of recently detected hematological abnormalities. His medical history was notable for a diagnosis of idiopathic thrombocytopenic purpura at 3 years of age. His family history revealed multiple cases of AML, with 11 affected individuals across four maternal generations, eight of whom had died from the disease at different ages (Figure 1).

**FIGURE 1:**
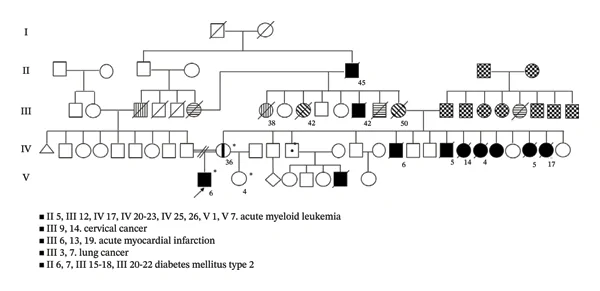
Family pedigree. This pedigree shows the segregation of acute myeloid leukemia, which affects 4 generations of the family, with an autosomal dominant inheritance pattern, the V‐1 index case with the confirmed germline mutation, the patient’s mother, IV‐10, an asymptomatic carrier with the same confirmed variant, and the healthy sister V‐2, who is not a carrier of the variant. In addition, several diseases that run in the family are shown, such as Type 2 diabetes mellitus, lung cancer, cervical cancer, and acute myocardial infarction.

The current illness began with constitutional symptoms, including hyporexia, asthenia, adynamia, and fever lasting 1 week. There was no known history of exposure to genotoxic agents. A complete blood count (CBC) performed one month before admission showed a hemoglobin level of 10.6 g/dL, hematocrit of 34.2%, mean corpuscular volume of 90 fL, leukocyte count of 2.53 × 10^3^/μL, lymphocyte count of 1.67 × 10^3^/μL, neutrophil count of 0.557 × 10^3^/μL, platelet count of 69 × 10^3^/μL, and no circulating blasts.

At admission, the CBC showed a leukocyte count of 3.9 × 10^3^/μL, lymphocyte count of 2.5 × 10^3^/μL, neutrophil count of 0.2 × 10^3^/μL, hemoglobin level of 9.5 g/dL, hematocrit of 30.7%, and platelet count 23 × 10^3^/μL. Peripheral blood examination showed 22% myeloblasts with Auer rods. The patient´s blood type was O Rh‐positive, and the direct antiglobulin (Coombs) test was negative.

Bone marrow aspiration showed 40% myeloblasts, consistent with AML. Immunophenotyping showed positivity for CD61, HLA‐DR, CD19, CD5, CD7, CD13++ (54%), CD117++ (55%), MPO++ (70%), and CD64. Notably, 31% of immature cells exhibited immunophenotypic features compatible with megakaryocytic differentiation. As part of the diagnostic workup, the patient was referred to the Medical Genetics department. Bone marrow cytogenetic analysis showed a normal male karyotype (46, XY) with no numerical or structural abnormalities. In addition, a multiplex RT‐PCR assay was negative for 28 common chromosomal translocations.

Molecular analysis by next‐generation sequencing (NGS) performed on peripheral blood using a 39‐gene panel for myelodysplastic syndromes identified a heterozygous CEBPA variant, c.273_309delinsGGCCAGGGTCT (p.Lys92Alafs∗7), classified as pathogenic. The variant is predicted to introduce a frameshift and a premature stop codon seven amino acids downstream of the indel site, resulting in a truncated protein with loss of the remaining 267 amino acids. Given the strong family history suggestive of an inherited predisposition to hematologic malignancy, first‐degree relatives underwent genetic testing. The patient’s mother carried the same heterozygous CEBPA variant, supporting its germline origin and autosomal dominant inheritance, whereas his sister did not carry the familial variant.

The patient received induction chemotherapy with cytarabine, dexrazoxane, daunorubicin, and etoposide over 6 weeks and achieved complete remission. He subsequently received a 39‐week treatment phase with cytarabine, mitoxantrone, and etoposide. During follow‐up, right axillary lymphadenopathy developed. Biopsy and histopathological examination of the lymph node showed follicular hyperplasia.

## 3. Discussion

The CEBPA gene encodes the transcription factor CCAAT/enhancer‐binding protein alpha and contains two alternative translation initiation sites that generate two major protein isoforms. The full‐length 42 kDa isoform (p42) consists of a 357‐amino acid protein and contains two N‐terminal transactivation domains (TAD1 and TAD2), as well as a C‐terminal basic leucine zipper (bZIP) domain involved in DNA binding and dimerization. An alternative translation initiation site at amino acid 120 generates a shorter 30 kDa isoform (p30). C/EBPα plays an important role in regulating cell proliferation and myeloid differentiation, in part through its interaction with the cell cycle regulators CDK2 and CDK4. An appropriate balance between the p42 and p30 is essential for normal myeloid differentiation [13–15].

Both somatic and germline CEBPA variants have been associated with AML, and pathogenic variants can disrupt normal C/EBPα function through truncation or alterations affecting critical functional domains [8, 12]. In our patient, we identified a heterozygous CEBPA variant c.273_309delinsGGCCAGGGTCT (p.Lys92Alafs∗7). This complex deletion‐insertion removes 37 nucleotides and introduces an 11‐nucleotide sequence (GGCCAGGGTCT), resulting in a frameshift and a premature termination codon 7 amino acids downstream of the indel site. The predicted consequence is a truncated protein of 99 amino acids. Because the reported CDK2/CDK4‐interacting region is located between amino acids 175 and 188, the predicted truncated p42 protein would lack this region [14]. In addition, because the second translation initiation site occurs downstream of the identified variant, translation of the p30 isoform could be preserved, providing a plausible mechanism for disrupting the normal p42/p30 balance and altering granulocyte differentiation [16]. This pathogenic variant has not been reported in patients with AML according to the Human Mutation database (https://www.hgmd.cf.ac.uk/ac/index.php).

The family history strongly suggests an autosomal dominant hereditary predisposition to AML. Eleven relatives across four maternal generations were reported to have AML, eight of whom died from the disease at different ages. Importantly, the patient’s mother carried the same heterozygous CEBPA variant but remained asymptomatic at the time of evaluation. Identification of the familial variant in both the patient and mother supports its germline origin and vertical transmission. The mother’s absence of clinical disease also illustrates that carrier status does not necessarily imply concurrent clinical manifestation and may reflect age‐dependent penetrance or variability in disease expression. Previous studies of familial AML associated with germline CEBPA variants have shown that acquisition of additional somatic CEBPA alterations can contribute to leukemogenesis [16, 17].

Previous reports indicate that AML associated with germline CEBPA variants may arise without a preceding documented phase of myelodysplasia or persistent cytopenia [17]. Our patient had previously been diagnosed with idiopathic thrombocytopenic purpura at 3 years of age. Although this finding raises the question of whether the earlier thrombocytopenia was related to the underlying predisposition, the available data are insufficient to establish such an association. Therefore, this antecedent should be interpreted cautiously. Nevertheless, a history of unexplained or recurrent cytopenias may warrant particular attention when evaluating pediatric patients with AML and a strong family history of hematologic malignancy.

Conventional cytogenetic and molecular studies did not identify an underlying abnormality in this patient: bone marrow karyotyping showed a normal chromosomal complement, and the multiplex RT‐PCR panel was negative for the 28 chromosomal translocations tested. These negative findings did not exclude an inherited predisposition. Subsequent NGS identified the CEBPA variant, and detection of the same variant in the patient’s mother supported its hereditary nature. This case therefore illustrates the potential value of incorporating germline predisposition assessment into the diagnostic evaluation of patients with AML when the personal or family history raises suspicion of an inherited hematologic malignancy syndrome.

The prognostic significance of CEBPA alterations depends on their molecular context. Favorable outcomes have been particularly associated with specific CEBPA mutational patterns, including bZIP in‐frame mutations [15]. The variant identified in our patient is a heterozygous N‐terminal frameshift and therefore should not automatically be assigned the prognostic implications described for other CEBPA molecular subgroups. Nevertheless, the patient achieved complete remission following chemotherapy and remains under clinical surveillance. Longer follow‐up will be necessary to characterize his clinical course and assess the durability of remission.

Identification of the familial CEBPA variant has important implications for genetic counseling. The patient’s mother, who carries the variant but is currently asymptomatic, requires appropriate clinical follow‐up, while the patient’s sister did not carry the family variant. Genetic evaluation of additional relatives could help further characterize segregation of the variant within the family and identify other at‐risk carriers. A major limitation of this report is the lack of genetic samples from deceased affected relatives, which prevents molecular confirmation of cosegregation between the variant and AML across the extended pedigree. Nevertheless, the multigenerational occurrence of AML, together with identification of the same CEBPA variant in the patient and his mother, supports an inherited predisposition in this family.

## 4. Conclusions

In this report, we present a previously unreported germline CEBPA deletion‐insertion variant in a family with a strong multigenerational history of AML. Identification of the same variant in the patient and his asymptomatic mother supports its germline origin and an autosomal dominant inheritance pattern. This case highlights the importance of considering an inherited predisposition to hematologic malignancy in pediatric patients with AML, particularly in the presence of a strong family history.

Our findings also illustrate the potential value of NGS in evaluating suspected hereditary predisposition to AML when conventional cytogenetic and molecular studies do not identify an underlying abnormality. Recognizing a germline predisposition has important implications not only for the affected patient but also for genetic counseling, identification of at‐risk relatives, and appropriate clinical surveillance.

Further studies, including functional characterization of the identified variant and assessment of additional somatic alterations and genetic evaluation of other affected and at‐risk family members, would help clarify this variant’s contribution to leukemogenesis and its segregation within the family. Long‐term follow‐up of asymptomatic carriers will also be important for better understanding the clinical expression of CEBPA‐associated familial AML.

## Author Contributions

Nancy Aislinn Tónix‐Ramírez: conceptualization and writing–original draft. Eneida Sánchez‐Medina: writing–review and editing. Luis Enrique Muñóz‐Pérez: writing–review and editing, Raquel Hernández‐Ramos: writing–original draft. Claudia Guevara‐Espejel: writing–review and editing. Elizabeth Bautista‐Rodríguez: writing–review and editing, Federico Osorio‐Antonio: conceptualization, writing–review and editing, and supervision. Federico Osorio‐Antonio had full access to all the data in this study and takes complete responsibility for the integrity of the data and the accuracy of the data analysis.

## Funding

This research did not receive any specific grant from funding agencies in the public, commercial, or not‐for‐profit sectors.

## Disclosure

All authors have read and approved the final version of the manuscript. A preprint of this manuscript was previously published in SSRN [Nancy Aislinn Tónix‐Ramirez, et al. 2025] available at: https://ssrn.com/abstract=5494716 [18].

## Consent

Written informed consent was obtained from the patient’s mother for the publication of the clinical information in this case report.

## Conflicts of Interest

The authors declare no conflicts of interest.

## Supporting Information

Additional supporting information can be found online in the Supporting Information section.

## Supporting information


**Supporting Information 1** Molecular findings for the case and relatives are available as Supporting Informations.

**Supporting Information 2** CARE‐checklist‐English‐2013.

## Data Availability

The data that support the findings of this study are available on Supporting Information 1.
