# Supplementary material for: Hereditary Predisposition to Acute Myeloid Leukemia: A Novel Germline CEBPA Mutation in a Multigenerational Family
Source: Case Rep Hematol. 2026 Sep 27;2026:8189036. doi: 10.1155/crh/8189036 (PMC13617314; doi:10.1155/crh/8189036)
Supplement: Supplementary file 1 [file CRH-2026-8189036-s001.pdf]

SUPPLEMENTARY INFORMATION

MOLECULAR ANALYSIS RESULT OF PATIENT

Sex: Male
Age: 6 years
Test performed: Sequence analysis and deletion/duplication testing of the 39 genes listed in the Genes Analyzed section, related to hereditary myelodysplastic syndrome/Leukemia Panel.

RESULT: POSITIVE

One Pathogenic variant identified in CEBPA. CEBPA is associated with autosomal dominant familial acute myeloid leukemia.

Additional Variant(s) of Uncertain Significance identified.

GENE	VARIANT	ZYGOSITY	VARIANT CLASSIFICATION
CEBPA	c.273_309delinsGGCCAGGGTCT (p.Lys92Alafs*7)	heterozygous	PATHOGENIC
BRCA2	c.121C>G (p.Pro41Ala)	heterozygous	Uncertain Significance

About this test

This diagnostic test evaluates 39 gene(s) for variants (genetic changes) that are associated with genetic disorders. Diagnostic genetic testing, when combined with family history and other medical results, may provide information to clarify individual risk, support a clinical diagnosis, and assist with the development of a personalized treatment and management strategy.

Clinical summary

A Pathogenic variant, c.273_309delinsGGCCAGGGTCT (p.Lys92Alafs*7), was identified in CEBPA.

- The CEBPA gene is associated with autosomal dominant predisposition to familial acute myeloid leukemia (AML) (MedGen UID: 9730).
- This result is consistent with a predisposition to, or diagnosis of, CEBPA-related conditions.
- Familial AML is characterized by multiple diagnoses of this cancer type within a family. The age at diagnosis can be variable; however, it appears to have an earlier onset than sporadic cases (PMID: 18946494, 26162409).
- Biological relatives have a chance of being at risk for CEBPA-related conditions and should consider testing if clinically appropriate.

A Variant of Uncertain Significance, c.121C>G (p.Pro41Ala), was identified in BRCA2.

- The BRCA2 gene is associated with autosomal dominant hereditary breast and ovarian cancer (HBOC) syndrome (MedGen UID: 151793) and autosomal recessive Fanconi anemia, type D1 (FA-D1) (MedGen UID: 325420).
- Not all variants present in a gene cause disease. The clinical significance of the variant(s) identified in this gene is uncertain. Until this uncertainty can be resolved, caution should be exercised before using this result to inform clinical management decisions.
- Complimentary familial VUS testing is not offered. Details on our VUS Resolution and Family Variant Testing Programs can be found at https://www.invitae.com/family.

Variant details

CEBPA, Exon 1, c.273_309delinsGGCCAGGGTCT (p.Lys92Alafs*7), heterozygous, PATHOGENIC

- This sequence change creates a premature translational stop signal (p.Lys92Alafs*7) in the CEBPA gene. While this is not anticipated to result in nonsense mediated decay, it is expected to disrupt the last 267 amino acid(s) of the CEBPA protein.
- This variant is not present in population databases (gnomAD no frequency).
- This variant has not been reported in the literature in individuals affected with CEBPA-related conditions.
- This variant disrupts the production of the full length isoform (p42) of the CEBPA protein, which results in the preferential usage of an alternate downstream in-frame methionine at codon 120. Translation starting from this methionine results in a 30 kDa N-terminal truncated isoform of CEBPA that lacks the TAD1 domain, and has been shown to abrogate CEBPA function by acting as a dominant-negative allele (PMID: 11242107, 19953636, 26162409). While functional studies have not been performed to directly test the effect of this variant on CEBPA protein function, this suggests that disruption of this region of the protein is causative of disease.
- This variant disrupts the region of the CEBPA protein between codon 15 and 119. Other variants in this region have been observed in individuals with autosomal dominant CEBPA-related conditions (PMID: 11242107, 31867767, 32430494; Invitae), which suggests that this may be a clinically significant region of the protein.
- For these reasons, this variant has been classified as Pathogenic.

BRCA2, Exon 3, c.121C>G (p.Pro41Ala), heterozygous, Uncertain Significance

- This sequence change replaces proline, which is neutral and non-polar, with alanine, which is neutral and non-polar, at codon 41 of the BRCA2 protein (p.Pro41Ala).
- This variant is present in population databases (rs80358415, gnomAD 0.006%).
- This variant has not been reported in the literature in individuals affected with BRCA2-related conditions.
- ClinVar contains an entry for this variant (Variation ID: 462228).
- Advanced modeling of protein sequence and biophysical properties (such as structural, functional, and spatial information, amino acid conservation, physicochemical variation, residue mobility, and thermodynamic stability) performed at Invitae indicates that this missense variant is not expected to disrupt BRCA2 protein function with a negative predictive value of 95%.
- In summary, the available evidence is currently insufficient to determine the role of this variant in disease. Therefore, it has been classified as a Variant of Uncertain Significance.

Genes analyzed

This table represents a complete list of genes analyzed for this individual, including the relevant gene transcript(s). If more than one transcript is listed for a single gene, variants were reported using the first transcript listed unless otherwise indicated in the report. An asterisk (*) indicates that this gene has a limitation. Please see the Limitations section for details. Results are negative unless otherwise indicated in the report. Benign and Likely Benign variants are not included in this report and in specific scenarios variants of uncertain significance in the requisitioned gene(s) may not be included in this report.

GENE	TRANSCRIPT
ANKRD26*	NM_014915.2
ATM*	NM_000051.3
BLM	NM_000057.3
BRCA1	NM_007294.3
BRCA2	NM_000059.3
CBL	NM_005188.3
CEBPA	NM_004364.4
CHEK2	NM_007194.3
DDX41	NM_016222.3
ELANE	NM_001972.2
EPCAM*	NM_002354.2
ERCC6L2	NM_020207.4
ETV6	NM_001987.4
G6PC3	NM_138387.3
GATA2	NM_032638.4
GFI1*	NM_005263.3
HAX1	NM_006118.3
IKZF1	NM_006060.6
KDM1A	NM_001009999.2
KRAS	NM_004985.4
MBD4	NM_003925.2
MECOM	NM_001105078.3
MLH1*	NM_000249.3
MSH2*	NM_000251.2
MSH6*	NM_000179.2
NBN	NM_002485.4
NF1*	NM_000267.3
PAX5	NM_016734.2
PMS2*	NM_000535.5
PTPN11	NM_002834.3
RTEL1	NM_001283009.1
RUNX1	NM_001754.4
SAMD9	NM_017654.3
SAMD9L	NM_152703.4
SRP72	NM_006947.3

GENE	TRANSCRIPT
TERC	NR_001566.1
TERT	NM_198253.2
TET2	NM_001127208.2
TP53	NM_000546.5

MOLECULAR ANALYSIS RESULT OF MOTHER PATIENT

Sex: Female
Age: 33 years
Test performed: Sequence analysis and deletion/duplication testing of the 2 genes listed in the Genes Analyzed section

REQUESTED VARIANTS

GENE	VARIANT	ZYGOSITY	VARIANT CLASSIFICATION	RESULT
CEBPA	c.273_309delinsGGCCAGGGTCT (p.Lys92Alafs*7)	heterozygous	PATHOGENIC	Detected
BRCA2	c.121C>G (p.Pro41Ala)	N/A	Uncertain Significance	Not detected

The table above reflects the information for the requested variant(s) as of the date that this report was issued. Please see the result box for a summary of any reportable findings.

RESULT: POSITIVE

One Pathogenic variant identified in CEBPA. CEBPA is associated with autosomal dominant familial acute myeloid leukemia.

GENE	VARIANT	ZYGOSITY	VARIANT CLASSIFICATION
CEBPA	c.273_309delinsGGCCAGGGTCT (p.Lys92Alafs*7)	heterozygous	PATHOGENIC

About this test

This diagnostic test evaluates 2 gene(s) for variants (genetic changes) that are associated with genetic disorders. Diagnostic genetic testing, when combined with family history and other medical results, may provide information to clarify individual risk, support a clinical diagnosis, and assist with the development of a personalized treatment and management strategy.

Clinical summary

A Pathogenic variant, c.273_309delinsGGCCAGGGTCT (p.Lys92Alafs*7), was identified in CEBPA.

- The CEBPA gene is associated with autosomal dominant predisposition to familial acute myeloid leukemia (AML) (MedGen UID: 9730).
- This result is consistent with a predisposition to, or diagnosis of, CEBPA-related conditions.
- Familial AML is characterized by multiple diagnoses of this cancer type within a family. The age at diagnosis can be variable; however, it appears to have an earlier onset than sporadic cases (PMID: 18946494, 26162409).
- Biological relatives have a chance of being at risk for CEBPA-related conditions and should consider testing if clinically appropriate.

Variant details

CEBPA, Exon 1, c.273_309delinsGGCCAGGGTCT (p.Lys92Alafs*7), heterozygous, PATHOGENIC

- This sequence change creates a premature translational stop signal (p.Lys92Alafs*7) in the CEBPA gene. While this is not anticipated to result in nonsense

mediated decay, it is expected to disrupt the last 267 amino acid(s) of the CEBPA protein.

- This variant is not present in population databases (gnomAD no frequency).
- This variant has not been reported in the literature in individuals affected with CEBPA-related conditions.
- This variant disrupts the production of the full length isoform (p42) of the CEBPA protein, which results in the preferential usage of an alternate downstream in-frame methionine at codon 120. Translation starting from this methionine results in a 30 kDa N-terminal truncated isoform of CEBPA that lacks the TAD1 domain, and has been shown to abrogate CEBPA function by acting as a dominant-negative allele (PMID: 11242107, 19953636, 26162409). While functional studies have not been performed to directly test the effect of this variant on CEBPA protein function, this suggests that disruption of this region of the protein is causative of disease.
- This variant disrupts the region of the CEBPA protein between codon 15 and 119. Other variants in this region have been observed in individuals with autosomal dominant CEBPA-related conditions (PMID: 11242107, 31867767, 32430494; Invitae), which suggests that this may be a clinically significant region of the protein.
- For these reasons, this variant has been classified as Pathogenic.

Genes analyzed

This table represents a complete list of genes analyzed for this individual, including the relevant gene transcript(s). If more than one transcript is listed for a single gene, variants were reported using the first transcript listed unless otherwise indicated in the report. An asterisk (*) indicates that this gene has a limitation. Please see the Limitations section for details. Results are negative unless otherwise indicated in the report. Benign and Likely Benign variants are not included in this report and in specific scenarios variants of uncertain significance in the requisitioned gene(s) may not be included in this report.

GENE	TRANSCRIPT
BRCA2	NM_000059.3

GENE	TRANSCRIPT
CEBPA	NM_004364.4

MOLECULAR ANALYSIS RESULT OF SISTER PATIENT

Sex: Female
Age: 4 years
Test performed: Sequence analysis and deletion/duplication testing of the 2 genes listed in the Genes Analyzed section.

REQUESTED VARIANTS

GENE	VARIANT	ZYGOSITY	VARIANT CLASSIFICATION	RESULT
BRCA2	c.121C>G (p.Pro41Ala)	N/A	Uncertain Significance	Not detected
CEBPA	c.273_309delinsGGCCAGGGTCT (p.Lys92Alafs*7)	N/A	PATHOGENIC	Not detected

The table above reflects the information for the requested variant(s) as of the date that this report was issued. Please see the result box for a summary of any reportable findings.

RESULT: NEGATIVE

About this test

This diagnostic test evaluates 2 gene(s) for variants (genetic changes) that are associated with genetic disorders. Diagnostic genetic testing, when combined with family history and other medical results, may provide information to clarify individual risk, support a clinical diagnosis, and assist with the development of a personalized treatment and management strategy.

Clinical summary

No reportable genetic variants were identified by this analysis; however, this individual may still be at risk for certain medical conditions based on other factors such as family history, genetic causes not evaluated with this test, or other environmental influences. Follow-up of this individual and surveillance of their family members may still be indicated.

Genes analyzed

This table represents a complete list of genes analyzed for this individual, including the relevant gene transcript(s). If more than one transcript is listed for a single gene, variants were reported using the first transcript listed unless otherwise indicated in the report. An asterisk (*) indicates that this gene has a limitation. Please see the Limitations section for details. Results are negative unless otherwise indicated in the report. Benign and Likely Benign variants are not included in this report and in specific scenarios variants of uncertain significance in the requisitioned gene(s) may not be included in this report.

GENE	TRANSCRIPT
BRCA	NM_000059.3

GENE	TRANSCRIPT
CEBP	NM_004364.4

Methods

- Genomic DNA obtained from the submitted sample is enriched for targeted regions using a hybridization-based protocol, and sequenced using Illumina technology. Unless otherwise indicated, all targeted regions are sequenced with ≥50x depth or are supplemented with additional analysis. Reads are aligned to a reference sequence (GRCh37), and sequence changes are identified and interpreted in the context of a single clinically relevant transcript, indicated in the Genes Analyzed table. Enrichment and analysis focus on the coding sequence of the indicated transcripts, 20bp of flanking intronic sequence, and other specific genomic regions demonstrated to be causative of disease at the time of assay design. Promoters, untranslated regions, and other non-coding regions are not otherwise interrogated. For some genes only targeted loci are analyzed. Exonic deletions and duplications are called using an in-house algorithm that determines copy number at each target by comparing the read depth for each target in the proband sequence with both mean read-depth and read-depth distribution, obtained from a set of clinical samples. Markers across the X and Y chromosomes are analyzed for quality control purposes and may detect deviations from the expected sex chromosome complement. Such deviations may be included in the report in accordance with internal guidelines. Variants are reported according to the Human Genome Variation Society (HGVS) guidelines. Confirmation of the presence and location of reportable variants is performed as needed based on stringent criteria using one of several validated orthogonal approaches (PubMed ID 30610921). Sequencing is performed by Invitae Corporation (1400 16th Street, San Francisco, CA 94103, #05D2040778). Confirmatory sequencing is performed by Invitae Corporation (1400 16th Street, San Francisco, CA 94103, #05D2040778). RNA sequencing is performed by Invitae Corporation (1400 16th Street, San Francisco, CA 94103, #05D2040778).

  The following additional analyses are performed if relevant to the requisition. For PMS2 exons 12-15, the reference genome has been modified to force all sequence reads derived from PMS2 and the PMS2CL pseudogene to align to PMS2, and variant calling algorithms are modified to support an expectation of 4 alleles. If a rare SNP or indel variant is identified by this method, both PMS2 and the PMS2CL pseudogene are amplified by long-range PCR and the location of the variant is determined by Pacific Biosciences (PacBio) SMRT sequencing of the relevant exon in both long-range amplicons. If a CNV is identified, MLPA or MLPA-seq is run to confirm the variant. If confirmed, both PMS2 and PMS2CL are amplified by long-range PCR, and the identity of the fixed differences between PMS2 and PMS2CL are sequenced by PacBio from the long-range amplicon to disambiguate the location of the CNV. For C9orf72 repeat expansion testing, hexanucleotide repeat units are detected by repeat-primed PCR (RP-PCR) with fluorescently labeled primers followed by capillary electrophoresis. Interpretation Reference Ranges: Benign (Normal Range): <25 repeat units, Uncertain: 25-30 repeat units, Pathogenic (Full Mutation): >=31 repeat units (PMID: 21944779, 22406228, 23111906, 28689190, 31315673, 33168078, 33575483). A second round of RP-PCR utilizing a non-overlapping set of primers is used to confirm the initial call in the case of suspected allele sizes of 22 or more repeats. For RNA analysis of the genes indicated in the Genes Analyzed table, complementary DNA is synthesized by reverse transcription from RNA derived from a blood specimen and enriched for specific gene sequences using capture hybridization. After high-throughput sequencing using Illumina technology, the output reads are aligned to a reference sequence (genome build GRCh37; custom derivative of the RefSeq transcriptome) to identify the locations of exon junctions through the detection of split reads. The relative usage of exon junctions in a test specimen is assessed quantitatively and compared to the usage seen in control specimens. Abnormal exon junction usage is evaluated as evidence in the Sherloc variant interpretation framework. If an abnormal splicing pattern is predicted based on a DNA variant outside the typical reportable range, as described above, the presence of the variant is confirmed by targeted DNA sequencing.

- A PMID is a unique identifier referring to a published, scientific paper. Search by PMID at **http://www.ncbi.nlm.nih.gov/pubmed**.
- An rsID is a unique identifier referring to a single genomic position, and is used to associate population frequency information with sequence changes at that position. Reported population frequencies are derived from a number of public sites that aggregate data from large-scale population sequencing projects, including ExAC (**http://exac.broadinstitute.org**), gnomAD (**http://gnomad.broadinstitute.org**), and dbSNP (**http://ncbi.nlm.nih.gov/SNP**).
- A MedGen ID is a unique identifier referring to an article in MedGen, NCBI's centralized database of information about genetic disorders and phenotypes. Search by MedGen ID at **http://www.ncbi.nlm.nih.gov/medgen**. An OMIM number is a unique identifier referring to a comprehensive entry in Online Mendelian Inheritance in Man (OMIM). Search by OMIM number at **http://omim.org/**.
- Invitae uses information from individuals undergoing testing to inform variant interpretation. If "Invitae" is cited as a reference in the variant details this may refer to the individual in this requisition and/or historical internal observations.

Limitations

Based on validation study results, this assay achieves >99% analytical sensitivity and specificity for single nucleotide variants, insertions and deletions <15bp in length, and exon-level deletions and duplications. Invitae's methods also detect insertions and deletions larger than 15bp but smaller than a full exon but sensitivity for these may be marginally reduced. Invitae's deletion/duplication analysis determines copy number at a single exon resolution at virtually all targeted exons. However, in rare situations, single-exon copy number events may not be analyzed due to inherent sequence properties or isolated reduction in data quality. Certain types of variants, such as structural rearrangements (e.g. inversions, gene conversion events, translocations, etc.) or variants embedded in sequence with complex architecture (e.g. short tandem repeats or segmental duplications), may not be detected.

Additionally, it may not be possible to fully resolve certain details about variants, such as mosaicism, phasing, or mapping ambiguity. Unless explicitly guaranteed, sequence changes in the promoter, non-coding exons, and other non-coding regions are not covered by this assay. Please consult the test definition on our website for details regarding regions or types of variants that are covered or excluded for this test. This report reflects the analysis of an extracted genomic DNA sample. While this test is intended to reflect the analysis of extracted genomic DNA from a referred patient, in very rare cases the analyzed DNA may not represent that individual's constitutional genome, such as in the case of a circulating hematolymphoid neoplasm, bone marrow transplant, blood transfusion, chimerism, culture artifact or maternal cell contamination. Interpretations are made on the assumption that any clinical information provided, including specimen identity, is accurate. Invitae's RNA analysis is not designed for use as a stand-alone diagnostic method and cannot determine absolute RNA levels. Results from the RNA analysis may not be informative for interpreting copy number events. Additionally, sensitivity to detect RNA splicing events may be reduced for variants in the first donor site of each gene.

ATM: Sequencing analysis for exons 6, 24, 43 includes only cds +/- 10 bp. EPCAM: Sequencing analysis is not offered for this gene. MLH1: Sequencing analysis for exons 12 includes only cds +/- 10 bp. MSH2: Analysis includes the exon 1-7 inversion (Boland mutation). Sequencing analysis for exons 2, 5 includes only cds +/- 10 bp. MSH6: Sequencing analysis for exons 7, 10 includes only cds +/- 10 bp. NF1: Sequencing analysis for exons 2, 7, 25, 41, 48 includes only cds +/- 10 bp. PMS2: Sequencing analysis for exons 7 includes only cds +/- 10 bp. GFI1: Sequencing analysis for exons 6 includes only cds +/- 0 bp. ANKRD26: Sequencing analysis for exons 19 includes only cds +/- 0 bp.
